# Increases in HIV Incidence Following Receptive Anal Intercourse Among Women: A Systematic Review and Meta-analysis

**DOI:** 10.1007/s10461-019-02651-0

**Published:** 2019-09-04

**Authors:** James Stannah, Romain Silhol, Jocelyn Elmes, Branwen Owen, Barbara L. Shacklett, Peter Anton, Ian McGowan, Ariane van der Straten, Dobromir Dimitrov, Rebecca F. Baggaley, Marie-Claude Boily

**Affiliations:** 1 MRC Centre for Global Infectious Disease Analysis, Department of Infectious Disease Epidemiology, Imperial College London, London, UK; 2 Department of Public Health, Environments and Society, London School of Hygiene & Tropical Medicine, London, UK; 3 Department of Medical Microbiology and Immunology, University of California, Davis, Davis, CA USA; 4 Center for HIV Prevention Research, University of California, Los Angeles, Los Angeles, CA USA; 5 School of Medicine, University of Pittsburgh, Pittsburgh, PA USA; 6 Women’s Global Health Imperative, RTI International, San Francisco, CA USA; 7 Vaccine and Infectious Disease Division, Fred Hutchinson Cancer Research Center, Seattle, WA USA; 8 HPTN Modelling Centre, Imperial College London, London, UK

**Keywords:** Anal intercourse, HIV, Heterosexual, Women, Sexual behaviour, Meta-analysis

## Abstract

Receptive anal intercourse (RAI) carries a greater per-act risk of HIV acquisition than receptive vaginal intercourse (RVI) and may influence HIV epidemics driven by heterosexual sex. This systematic review explores the association between RAI and incident HIV among women, globally. We searched Embase and Medline through September 2018 for longitudinal studies reporting crude (cRR) or adjusted (aRR) relative risks of HIV acquisition by RAI practice among women. Of 27,563 articles identified, 17 eligible studies were included. We pooled independent study estimates using random-effects models. Women reporting RAI were more likely to acquire HIV than women not reporting RAI (pooled cRR = 1.56 95% CI 1.03–2.38, N = 18, I^2^ = 72%; pooled aRR = 2.23, 1.01–4.92, N = 5, I^2^ = 70%). In subgroup analyses the association was lower for women in Africa (pooled cRR = 1.16, N = 13, I^2^ = 21%) than outside Africa (pooled cRR = 4.10, N = 5, I^2^ = 79%) and for high-risk (pooled aRR = 1.69, N = 4, I^2^ = 63%) than general-risk women (pooled aRR = 8.50, N = 1). Interview method slightly influenced cRR estimates (*p* value = 0.04). In leave-one-out sensitivity analyses pooled estimates were generally robust to removing individual study estimates. Main limitations included poor exposure definition, incomplete adjustment for confounders, particularly condom use, and use of non-confidential interview methods. More and better data are needed to explain differences in risk by world region and risk population. Women require better counselling and greater choice in prevention modalities that are effective during RVI and RAI.

**Electronic supplementary material:**

The online version of this article (10.1007/s10461-019-02651-0) contains supplementary material, which is available to authorized users.

## Introduction

HIV acquisition risk during one receptive anal intercourse (RAI) act unprotected by pre-exposure prophylaxis (PrEP) or condoms (URAI) is higher than during receptive vaginal intercourse (RVI) unprotected by PrEP or condoms (URVI), with pooled estimates from previous systematic reviews of 1.25% (95% confidence interval (CI) 0.55–2.23) and 0.08% (0.06–0.11) per URAI and URVI act, respectively [1–3]. Heterosexual URAI practice could increase HIV incidence among women and impact HIV spread at the population-level if practised sufficiently often by a sufficient fraction of women [4]. Current evidence from three systematic reviews among young women, South African women, and female sex workers (FSWs) suggests that RAI is common, with pooled estimates of the proportions practising RAI ranging from 12 to 21% in the past 3 months and from 15 to 22% in their lifetimes across these three risk populations [5–7]. Although scarcer and more heterogeneous, data on the frequency of anal intercourse suggest that 2–16% of all sex acts among all women in these different risk populations are RAI [6, 7].

Mathematical modelling studies suggest that given the elevated per-act HIV risk, HIV incidence among women who practise RAI for a period of time, even if infrequently, could be substantially higher than among women who do not [4, 8], with obvious implications for HIV spread and HIV prevention [9, 10]. For example, a risk equation model among high-risk women from 20 US cities estimated that 38% of HIV infections were due to RAI [11]. Another modelling analysis in Papua New Guinea predicted that if 20% of all women practised RAI in 10% of sex acts, and 90% of RAI acts were condomless, the total number of new infections would be 40% greater than if only RVI occurred [8].

However, empirical estimates from longitudinal studies have found mixed results with, for example, studies reporting HIV incidences 3.5 times greater among women reporting RAI [12], 6.4 times greater among women reporting URAI [13], or reporting no statistical association [14, 15]. To date, no studies have systematically reviewed the published evidence of an association between RAI or URAI and HIV incidence among women from longitudinal studies.

We aimed to (1) conduct the first systematic review of longitudinal studies reporting the association between RAI and HIV incidence among women globally, (2) produce pooled estimates of this association, (3) explore potential sources of heterogeneity across study estimates, and (4) test the robustness of pooled estimates to single study estimates.

## Methods

This systematic review and meta-analysis was conducted in accordance with MOOSE and PRISMA guidelines [16, 17].

### Search Strategy and Selection Criteria

We searched for longitudinal studies reporting estimates, or sufficient data to derive them, of the association between RAI over various recall periods and incident HIV, among women. Ovid Embase and Medline were searched for articles published between 1st January 1980 and 3rd September 2018 using terms for longitudinal study designs, women, sexual behaviour and HIV (see Supplementary Material for full search terms). Full-text articles were retrieved if abstracts reported heterosexual sexual behaviour and HIV and were screened for estimates of the association between RAI and incident HIV, and non-English articles were excluded. Reports from randomised controlled trials (RCTs), cohort studies and serodiscordant couple studies were included. We excluded cross-sectional studies, case–control studies, and reviews. Bibliographies of relevant articles were examined for additional references. Authors were also contacted for additional estimates (see Supplementary Material for details).

We extracted crude and adjusted estimates, or the necessary data to derive estimates (details in Supplementary Material) of the relative risk (RR) of the association between RAI exposure and HIV incidence, which was measured differently across studies. Studies either reported hazard rate ratios (HRR), incidence rate ratios (IRR), cumulative incidence ratios (CIR), or odds ratios (OR) of the RAI-HIV association. These different measures can produce slightly different estimates of the magnitude of the association under specific conditions. HRR (derived from survival analysis models) and IRR (based on HIV incidence per person-year) are expected to produce similar estimates [18]. However, CIR, OR, and HRR/IRR based estimates may differ if HIV incidence is high, follow-up duration is long, or if the magnitude of the association is large [19, 20]. Thus, to maximise uniformity across studies, HRR and IRR were extracted preferentially to CIR and OR, when available. When crude IRR was not directly reported but could be derived from the available data, derived crude IRR estimates were included preferentially to reported or derived CIR and OR. Crude HRR and adjusted HRR, IRR, CIR and OR could not be derived from published information. In situations where it was not possible to include HRR/IRR estimates, we included reported or derived CIR or OR estimates in order to maximise the number of included study estimates and assessed the influence of the type of measure on pooled estimates in subgroup analysis as described below.

Information on participant characteristics (e.g. world region, risk population, the percentage of study participants reporting RAI (the RAI prevalence), the frequency/number of RAI acts, antiretroviral treatment (ART) use by partner), study characteristics (e.g. study years, the recall period of RAI, definition of RAI) and study quality indicators (e.g. study design, interview method, whether the RR estimate was directly reported or self-calculated) was also extracted.

If multiple articles reported on the same study, the estimate from the largest sample was included. If articles reported multiple estimates from independent samples, all were included. If articles reported multiple estimates for smaller subgroups of a single sample, the combined estimate or largest subgroup was included. Both trial arms of RCTs were combined into a single group, provided the treatment had no significant effect in the original study, otherwise only the placebo group was included.

### Data Analysis

From this point onwards, we use the term “relative risk” (RR) to generically refer to the different measures of association (HRR, IRR, CIR and OR). Independent study estimates of crude (cRR) and adjusted (aRR) relative risks and 95% CIs were log-transformed and pooled using DerSimonian-Laird random-effects models based on inverse-variance [21]. To maintain uniformity, the 95% CIs of all study estimates were recalculated based on the normal approximation before pooling. Pooled estimates and 95% CIs were then exponentiated back to give estimates on the original scale, which were displayed on forest plots. Heterogeneity across study estimates was assessed using I^2^ statistics [22].

Potential sources of heterogeneity due to participant and study characteristics, type of measure of association (HRR, IRR, OR, CIR), and study quality indicators were explored in subgroup analyses on all variables and differences between subgroups were tested using Wald-type tests. In addition, we attributed scores reflecting study quality to each study estimate based on the Newcastle–Ottawa Scale (NOS) that varied from 0 to 9 points for estimates based on the lowest to highest quality studies, respectively [23]. Subgroup analysis was also used to explore publication bias stratifying by the location in the article where RAI was first mentioned (title/abstract, text/table) as studies may more prominently report stronger associations, and by whether RR estimates were self-derived or retrieved from contacted authors, as studies may not report estimates showing no effect. Publication bias was further assessed using funnel plots. Leave-one-out sensitivity analyses were conducted to assess how each study estimate influenced pooled estimates.

All analyses were conducted, and figures made, in R version 3.4.5 using the “metafor” package [24, 25].

## Results

### Search Results

**Fig. 1:**
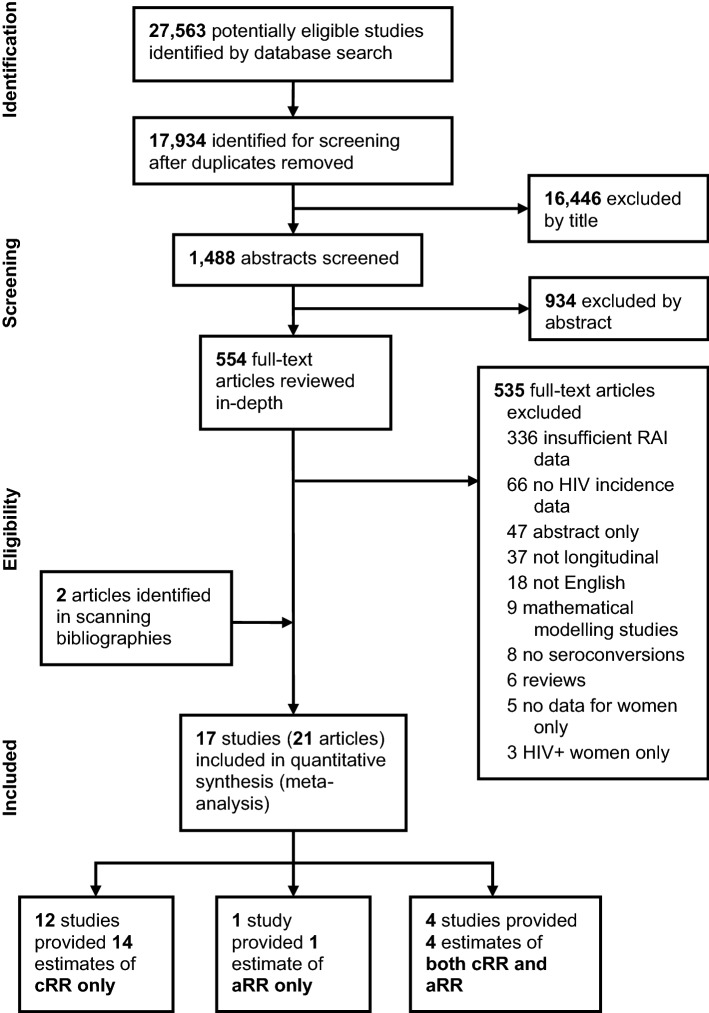
Study selection. The search identified 21 articles for inclusion in the meta-analysis reporting on 17 independent studies that provided crude and/or adjusted estimates of the relative risk of HIV acquisition associated with RAI among women. The search was conducted for articles published 1st January 1980 up to the 3rd September 2018

Database searches identified 27,563 titles, and 17,934 were screened after removing duplicates, resulting in 554 articles that went to full-text review (Fig. 1). Full-text review identified 19 articles and two further articles [12, 14] were identified through scanning bibliographies, giving a total of 21 included articles that reported on 17 unique longitudinal studies. Additionally, 18 authors were contacted, and one provided a single cRR estimate. None were able to provide further aRR estimates. In total, 16 studies reporting 18 independent cRR estimates (or sufficient data to derive estimates), including one study reporting three independent estimates, and five studies reporting five independent aRR estimates were included in the meta-analysis (Fig. 1). Two of the 21 articles were excluded from the analysis because they reported cRR and aRR estimates already reported in other articles [26, 27]. Four studies reported both cRR and aRR, 12 studies reported only cRR, and one study reported only aRR. Five of the 12 studies reporting only cRR estimates also conducted multivariate analyses but did not include RAI in the model.

### Study Characteristics

The main characteristics of the 17 studies included in the meta-analysis are presented in Table 1 (see Table S1 for additional details on included studies). Most studies were conducted in Africa (number of studies [N_s_] = 12) [12, 14, 15, 28–37] after ART was introduced in 1996 (N_s_ = 11) [13–15, 28–34, 36, 37], and the most common study design was cohort (N_s_ = 9) [28, 29, 31, 34–39], then RCT (N_s_ = 6) [12–15, 30, 32, 33], and serodiscordant couple studies (N_s_ = 2) [40–42]. Most studies were among high-risk women (N_s_ = 11), including FSWs (N_s_ = 6) [12, 14, 29, 31, 35, 37, 38], and other high-risk populations (N_s_ = 5) [13, 32, 33, 40–42] such as serodiscordant couples (SDCs) and high-risk HIV-negative women and the mean or median age in most studies was less than 28 years (N_s_ = 10) [12, 13, 28, 29, 32, 33, 35–37, 40, 41]. Study sample sizes varied hugely, ranging from 73 to 8859 women, and length of follow-up ranged from 258 to 8024 person-years (N_s_ = 15). Almost all studies recorded sexual behaviour data, including RAI, in face-to-face-interviews (FTFI) (N_s_ = 16) [12–15, 28–33, 35–42] and only one [34] (which reported three independent estimates) used audio computer-assisted self-interviews (ACASI) [43]. RAI was most commonly measured during follow-up (N_s_ = 10) [12, 15, 29, 30, 33, 34, 38–42] in the past 1 month (N_s_ = 4) [12, 29, 30], 3 months (N_s_ = 4) [15, 33, 34, 38], 6 months (N_s_ = 2) [40–42] and past year (N_s_ = 1) [39], and only four of these studies analysed RAI as a time-varying covariate [33, 34, 38, 42]. RAI was also measured at baseline (N_s_ = 7) [13, 14, 28, 31, 32, 35–37], ‘ever’ (N_s_ = 4) [13, 31, 35, 36] and in the past 1 month (N_s_ = 2) [28, 32]. Two articles reporting on the same study did not report the time frame of baseline RAI practise [14, 37]. Lifetime RAI prevalence ranged from 2 to 43%. RAI prevalence in the past 6 months ranged from 7 to 16%, 3 months from 2 to 15%, and 1 month from 2 to 42%. Most studies first mentioned RAI in the main text (N_s_ = 13) [12, 14, 15, 28, 30–38, 42], with five mentioning it first in the abstract or title [13, 29, 39–41]. No studies reported RAI frequency data. Two studies reporting only cRRs defined exposure as URAI only [13, 15]. A third study [41] controlled for condom use by dividing women into subgroups that ‘always’ or ‘sometimes/never’ used condoms for all sex acts, reporting a cRR estimate for the ‘sometimes/never’ subgroup only, as no seroconversions occurred in the ‘always’ subgroup. All other studies either specified inconsistent condom use during RAI or did not specify condom use. No included studies reported estimates of partner ART use.

**Table 1:** Table of included studies

Study	Study name (if applicable)	Study years	Study design	Interview method	World region	Risk population	Sample size	Follow-up (person-years)	Median age [Mean]	RAI prevalence (%)	Measurement of RAI	Definition of RAI	Location RAI first reported	Type of measure	cRR (95% CI)	aRR (95% CI)	Estimate included in meta-analysis
*Africa*																	
Laga et al. [35]	N/A	1988–1990	Cohort	FTFI	Africa	FSW	431	778	[25.8]	14.4	Baseline: Ever	Any RAI	Text	CIR	0.80^a^ (0.42–1.52)	NR	Yes, cRR
Ghys et al. [12]	N/A	1994–1998	RCT	FTFI	Africa	FSW	257	318	27	6.2	During follow-up: 1 month	Any RAI	Text	IRR	3.50 (1.01–12.11)	NR	Yes, cRR
Ramjee et al. [37]; Auvert et al. [14]	COL-1492	1996–2000	Cohort	FTFI	Africa	FSW	187	368	24 [25]	41.7	Baseline: Unclear	Any RAI	Text	HRR	NR	0.82 (0.46–1.44)	Yes, aRR
			RCT				88	NR	24 [28.7]^b^	43.2		Any RAI	Text	HRR	0.53 (0.22–1.29)	0.35 (0.12–1.00)	Yes, cRR only
Naicker et al. [31]	CAPRISA 002	2004–2007	Cohort	FTFI	Africa	FSW/high-risk^c^	242	390	[34.3]	34.3	Baseline: Ever	Any RAI	Text	HRR	1.49 (0.68–3.29)	1.65 (0.73–3.74)	Yes, both
Skoler-Karpoff et al. [15];	Carraguard Study	2004–2007	RCT	FTFI	Africa	General	6003	8024	[30.7]	2.1	During follow-up: 3 months	URAI only	Table	CIR	1.66^a^ (0.90–3.04)	NR	Yes, cRR
Wand and Ramjee [26]			Cohort				1456	1833	[32.5]^b^	5.5		URAI only	Text	CIR	1.30 (0.68–2.70)	NR	No
Watson-Jones et al. [33]	N/A	2004–2006	RCT	FTFI	Africa	High-risk HIV negative	821	1477	28 [27.9]^b^	NR	During follow-up: 3 months (time-varying)	Any RAI	Table	HRR	6.14^d^ (0.84–44.92)	6.87 (0.94–50.45)	Yes, both
Feldblum et al. [32]	SAVVY (Nigeria), SAVVY (Ghana), CS (Nigeria), CS (Multi-Country)	2004–2007	RCT	FTFI	Africa (9 sites), SE Asia (1 site)	High-risk HIV negative	7364	5486	[24.6]	NR	Baseline: 1 month	Any RAI	Table	HRR	1.48 (0.64–3.38)	NR	Yes, cRR
McCormack [30]	MDP301	2005–2008	RCT	FTFI	Africa	General	8859	7450	NR	2.0	During follow-up: 1 month	Any RAI	Table	IRR	1.45^a^ (0.79–2.64)	NR	Yes, cRR
Mavedzenge et al. [34]	MIRA Study	2003–2005	Cohort	ACASI	Africa (Durban)	General	1485	2193	[29.9]^b^	13.5	During follow-up: 3 months (time-varying)	Any RAI	Table	HRR	0.72 (0.34–1.55)	NR	Yes, cRR
					Africa (Harare)	General	2455	4197	[29.1]^b^	14.7				HRR	1.09 (0.50–2.39)	NR	Yes, cRR
					Africa (Jo’burg)	General	1008	1409	[30.2]^b^	15.3				HRR	0.88 (0.31–2.45)	NR	Yes, cRR
Nel et al. [36]	N/A	2007–2009	Cohort	FTFI	Africa	Family planning clinic	299	258	23 [24.1]	2.0	Baseline: Ever	Any RAI	Text	HRR	NR	8.50 (1.90–37.96)	Yes, aRR
Priddy et al. [29]	N/A	2008–2009	Cohort	FTFI	Africa	FSW	192	NR	[28]	35.4	During follow-up: 1 month	Any RAI	Title	IRR	0.45 (0.05–4.07)	NR	Yes, cRR
Dong et al. [28]	FRESH Study	2012–2016	Cohort	FTFI	Africa	General	945	512^a^	21	3.1	Baseline: 1 month	Any RAI	Table	CIR	0.77^a^ (0.11–5.41)	NR	Yes, cRR
*Out of Africa*																	
Saracco et al. [41]; Musicco et al. [27]; Saracco et al. [42]	Italian Partner Study	1987–1991	SDC	FTFI	Europe	SDC	134	222	[25.5]	15.7	During follow-up: 6 months	Any RAI	Abstract	IRR	1.40 (0.40–4.85)	NR	Yes, cRR
		1987–1992					436	638	[26.1]	15.4	During follow-up: 6 months (time-varying)	Any RAI	Text	HRR	NR	2.00 (0.80–4.80)	No
		1987–1996					627	1390	[28.3]	NR		Any RAI	Text	HRR	NR	2.50 (1.12–5.59)	Yes, aRR
de Vincenzi [40]	European Partner Study	1987–1991	SDC	FTFI	Europe	SDC	73	NR	[27.9]	11.0	During follow-up: 6 months	Any RAI	Abstract	IRR	1.20 (0.55–2.63)	NR	Yes, cRR
Kilmarx et al. [38]	Chiang Rai Health Club Study	1991–1996	Cohort	FTFI	SE Asia	FSW	285	695	NR	NR	During follow-up: 3 months (time-varying)	Any RAI	Text	HRR	24.30 (3.12–189.33)	NR	Yes, cRR
Chirgwin et al. [39]	N/A	1990–1993	Cohort	FTFI	Americas	Health clinic	449	664	30.2	14.5	During follow-up: 1 year	Any RAI	Abstract	OR	19.80 (1.25–313.94)	NR	Yes, cRR
Novak et al. [13]	Step Study	2005–2007	RCT	FTFI	Americas	High-risk HIV negative	621	1842^a^	27	11.9	Baseline: Ever	URAI only	Abstract	CIR	6.40 (3.81–10.74)	NR	Yes, cRR

*ACASI* audio computer-assisted self-interview, *aRR* adjusted relative risk, *CI* confidence interval, *CIR* cumulative incidence ratio, *cRR* crude relative risk, *FSW* female sex worker, *FTFI* face-to-face interview, *HIV* human immunodeficiency virus, *HRR* hazard rate ratio, *IRR* incidence rate ratio, *N/A* not applicable, *NR* not reported, *OR* odds ratio, *RAI* receptive anal intercourse, *RCT* randomised controlled trial, *SDC* serodiscordant couple, *SE Asia* Southeast Asia, *URAI* unprotected (condomless and no PrEP) receptive anal intercourse

^a^Estimate self-calculated (see Supplement for details)

^b^Mean age approximated from reported age ranges

^c^High-risk women in this study defined as women with at least 3 partners in the 3 months prior to recruitment

^d^Estimate provided by authors after being contacted

Most studies reported HRR (number of estimates [N_e_] = 7), then IRR (N_e_ = 6), CIR (N_e_ = 4), and OR (N_e_ = 1). Three crude IRR, three crude CIR, and their 95% CIs were self-derived. All five adjusted estimates were HRR. Two were adjusted for age only [33, 36] and one for herpes simplex virus-2 (HSV-2) infection only [37]. The remaining two were adjusted for age, sexual behaviour, sexually transmitted infections (STIs) [31]; and for condom use, symptoms of HIV/AIDS-related disease in the partner living with HIV, and ART use by the partner living with HIV [42]. One study reported a single crude HRR combining data from four microbicide trials conducted in nine sites in Africa and one site in India [32], and was therefore included as an African estimate in subgroup analyses.

All estimates received a NOS score between 5 and 8, with 6 the most common score (N_e_ = 13) [13, 15, 28, 30, 32, 33, 35–41], indicating most studies were of adequate quality (Table S2). Across all estimates the most commonly failed criteria was ascertainment of exposure using FTFI rather than more confidential methods. For cRR, failure to adjust for potential confounders and for aRR, failure to adjust for important confounders including condom use, produced lower scores. Only one aRR estimate was adjusted for condom use.

### Does RAI Practise Increase the Risk of HIV Acquisition Among Women?

**Fig. 2:**
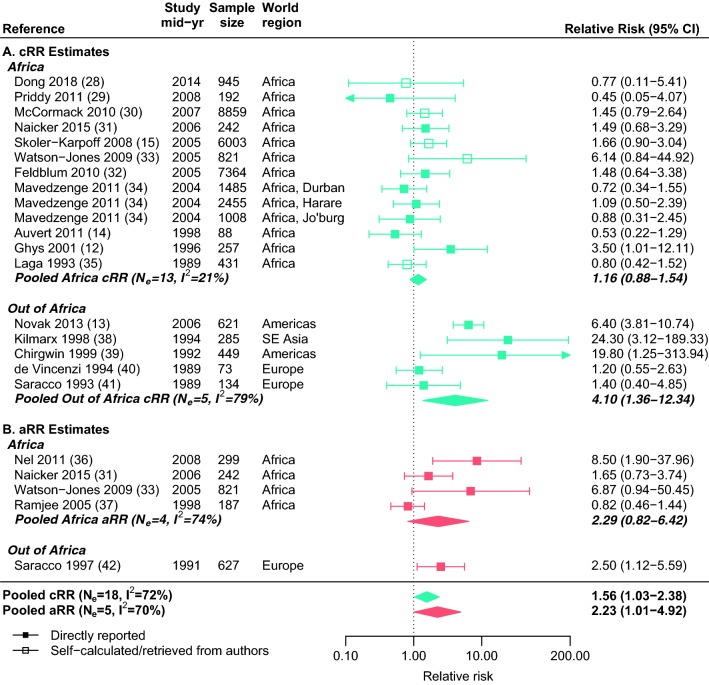
Forest plot of crude and adjusted study and pooled estimates. Crude (cRR, blue) and adjusted (aRR, red) study estimates of relative risk (squares), and corresponding pooled estimates (diamonds) of the association between HIV incidence and receptive anal intercourse (RAI) among women are given overall and stratified by world region. The dotted vertical line represents a relative risk of 1 (i.e. no effect of RAI on HIV incidence) (Color figure online)

The meta-analysis included 18 independent cRR estimates ranging from 0.45 to 24.3 across a total of 31,712 women (Fig. 2a), and five independent aRR estimates ranging from 0.82 to 8.50 across a total of 2176 women (Fig. 2b). Despite substantial heterogeneity across estimates, the pooled cRR (1.56, 95% CI 1.03–2.38, I^2^ = 72%, N = 18) and aRR (2.23, 95% CI 1.01–4.92, I^2^ = 70%, N = 5) suggested significantly higher HIV incidence rates among women reporting RAI (Fig. 2).

In the subset of four studies reporting both crude and adjusted estimates, the individual cRR and aRR estimates did not differ hugely (average difference = 39%) and the pooled cRR and aRR were of similar magnitude (pooled cRR = 1.26, 95% CI 0.57–2.80, N = 4; pooled aRR = 1.69, 95% CI 0.82–3.47, N = 4) (Fig. S1) to the overall pooled estimates based on all study estimates.

### Subgroup Analysis

#### How Do Characteristics of the Study Participants Influence the RR?

In subgroup analyses, crude study estimates varied by world region (p = 0.03) with higher pooled estimates for studies outside Africa (pooled = 4.10, 95% CI 1.36–12.3, N_e_ = 5, I^2^ = 79%) than in Africa (pooled = 1.16, 95% CI 0.88–1.54, N_e_ = 13, I^2^ = 21%) (Fig. 2a, Table 2a). The small number of studies limited exploration of the heterogeneity across adjusted study estimates. Pooled aRR did not differ by world region (p = 0.90) but differed slightly (although non-significantly) by risk populations (p = 0.06) (Fig. 2, Table S3), with the pooled aRR for general-risk women higher (pooled = 8.50, 95% CI 1.90–38.0, N_e_ = 1) than for high-risk women (pooled = 1.69, 95% CI 0.82–3.47, N_e_ = 3, I^2^ = 63%) (Table S3). Pooled cRR and aRR did not differ significantly by other participant characteristics, including mean age or RAI prevalence.

**Table 2:** Subgroup analyses of crude study estimates (cRR) stratified by participant and study characteristics and quality indicators

Variable	N_e_	References	Pooled cRR	95% CI	p value	I^2^ (%)
a. Participant characteristics						
World region					**0.03**	
Africa	13	[12, 14, 15, 28–35]	1.16	0.88–1.54	–	21
Out of Africa	5	[13, 38–41]	4.10	1.36–12.3	–	79
Risk population					0.27	
General-risk	7	[15, 28, 30, 34, 41]	1.22	0.84–1.78	–	22
High-risk	11	[12–14, 29, 31–33, 35, 38–40]	1.85	0.97–3.51	–	79
Mean age (missing = 6)					0.76	
≤ 28	6	[29, 32, 33, 35, 39, 40]	1.14	0.77–1.69	–	2
> 28	6	[14, 15, 31, 34]	1.05	0.73–1.50	–	20
RAI prevalence (missing = 3)					0.14	
≤ 14%	8	[12, 13, 15, 28, 30, 34, 39]	1.77	0.91–3.44	–	81
> 14%	7	[14, 29, 31, 34, 35, 40, 41]	0.96	0.62–1.49	–	29
b. Study characteristics and quality indicators						
Study year^a^					0.32	
Pre-1996	6	[35, 38–41]	2.30	0.96–5.48	–	69
1996 onwards	12	[12–15, 28–34]	1.37	0.83–2.27	–	75
Study design					0.50	
Cohort	9	[28, 29, 31, 34, 35, 38, 41]	1.20	0.70–2.05	–	52
RCT	7	[12–15, 30, 32, 33]	2.05	1.03–4.10	–	81
Serodiscordant couple	2	[39, 40]	1.25	0.65–2.43	–	0
Interview method					**0.04**	
ACASI	3	[34]	0.88	0.54–1.43	–	0
FTFI	15	[12–15, 28–33, 35, 38–41]	1.81	1.11–2.94	–	73
Measurement of exposure^b^					0.73	
Baseline: short time frame	2	[28, 32]	1.34	0.62–2.88	–	0
Baseline: long time frame	3	[13, 31, 35]	2.00	0.52–7.67	–	92
During follow-up: anytime	7	[12, 15, 29, 30, 39–41]	1.58	1.09–2.29	–	10
During follow-up: time-varying	5	[33, 35, 38]	1.70	0.68–4.26	–	69
Unclear	1	[14]	0.53	0.22–1.29	–	–
Definition of RAI					0.17	–
URAI only	2	[13, 15]	3.29	0.88–12.4	–	91
Any RAI	16	[12, 14, 28–35, 38–41]	1.27	0.90–1.79	–	43
Type of measure					0.38	
HRR	8	[14, 32–34, 38]	1.28	0.75–2.20	–	58
IRR	5	[12, 29, 30, 39, 40]	1.45	0.96–2.19	–	0
CIR	4	[13, 15, 28, 35]	1.77	0.58–5.36	–	89
OR	1	[41]	19.8	1.25–314	–	–
Extraction of estimate					0.53	
Directly reported	13	[12–14, 29, 31, 32, 34, 38–41]	1.67	0.94–2.96	–	77
Self-calculated/from authors	5	[15, 28, 30, 33, 35]	1.32	0.85–2.05	–	28
Location RAI first reported					0.32	
Title/abstract	5	[13, 29, 39–41]	2.33	0.76–7.09	–	79
Text/table	13	[12, 14, 15, 28, 30–35, 38]	1.29	0.91–1.82	–	47
NOS score					0.33	
5	5	[12, 13, 29, 31, 33]	1.42	0.59–3.38	–	59
6	10	[13, 15, 28, 30, 32, 35, 38–41]	2.01	1.11–3.64	–	78
7	3	[34]	0.88	0.54–1.43	–	0

*ACASI* Audio computer-assisted self-interview, *CI* confidence interval, *CIR* cumulative incidence ratio, *cRR* crude relative risk, *FTFI* face-to-face interview, *IRR* incidence rate ratio, *HRR* hazard rate ratio, *N*_*e*_ number of estimates, *NOS* Newcastle–Ottawa Scale, *NR* not reported, *OR* odds ratio, *RAI* receptive anal intercourse, *RCT* randomised controlled trial, *URAI* unprotected (condomless and no PrEP) receptive anal intercourse

Continuous variables were dichotomised at the median, except for study year, which was dichotomised at the boundary between the pre-, and post-antiretroviral treatment (ART) eras (1996). Statistically significant p-values are given in bold

^a^Study year is the midpoint between study start and finish

^b^Short time frame includes RAI in the past 6 months or less, long time frame includes RAI in the past year to lifetime

#### How Do Study Characteristics and Study Quality Influence the RR?

In subgroup analysis, pooled cRR only differed significantly by interview method (p = 0.04), with lower estimates in studies using ACASI (pooled = 0.88, 95% CI 0.54–1.43, N_e_ = 3, I^2^ = 0%) than FTFI (pooled = 1.81, 95% CI 1.11–2.94, N_e_ = 15, I^2^ = 73%) (Table 2b). Pooled cRR and aRR did not differ by other study characteristics or quality indicators, including study year, study design, measurement of exposure, definition of RAI, type of measure, and NOS score. In exploring potential publication bias, pooled cRR from study estimates directly reported in the original studies was slightly higher than when self-derived or retrieved from authors and from studies that more prominently reported RAI in the abstract or title rather than the main text (Table 2b). However, these differences were not statistically significant (Table 2b). Similar analyses could not be done for aRR estimates. Funnel plots also showed no evidence of publication bias across cRR estimates (Fig. S2A), but some evidence across aRR estimates (Fig. S2B).

#### How Do Individual Study Estimates Influence Pooled Estimates?

In leave-one-out sensitivity analyses, the direction of the associations remained intact (Fig. S3A–C). Overall, the pooled cRR estimate was mainly influenced by Novak’s [13] estimate among women in the US, one of the two studies defining RAI as URAI only (Fig. S3A). Omitting this estimate slightly lowered the pooled cRR and reduced the I^2^ value (pooled without Novak = 1.30, 95% CI 0.95–1.77, I^2^ = 42%) (Fig. S3A). Consistent with the low heterogeneity across study estimates, the pooled cRR for African studies was not influenced by any specific estimate (Fig. S3B). The overall pooled aRR was equally sensitive to most study estimates, which slightly influenced results in either direction, although omission of Ramjee substantially reduced the I^2^ value (I^2^ = 34%) (Fig. S3C).

## Discussion

Our review and meta-analysis of published longitudinal studies provides new knowledge on a key HIV acquisition risk among women who have sex with men. Overall, HIV incidence was approximately twice as high (pooled crude RR = 1.56, pooled adjusted RR = 2.23) among women reporting RAI than women reporting RVI only. Since women do not typically practise RAI in all sex acts, our pooled estimate is consistent with current evidence suggesting that HIV risk per URAI act is up to 10–20 times higher than per URVI act [1–3].

Our results also suggested a more modest RAI-HIV association for higher-risk populations and women in Africa, but more data are needed to fully explain these differences. Additionally, although our analysis suggested slight differences by world region, interview method, and risk population, all confidence intervals were wide and overlapping. Nonetheless, our results have implications for understanding HIV spread, identifying HIV interventions needs, and developing more HIV prevention modalities effective during both RVI and RAI [10, 44, 45].

To our knowledge, this is the first systematic review and meta-analysis on the increased risk of HIV acquisition through RAI compared to RVI among heterosexual women from longitudinal studies only and our study has several strengths that improve the generalisability and robustness of our findings. We used broad search terms to maximise coverage and restricted inclusion to longitudinal studies, ensuring that timings of RAI exposure and HIV acquisition were more precisely ascertained. We limited reporting and publication biases by deriving estimates which were not explicitly reported in publications and by contacting authors for additional estimates. Furthermore, we systematically extracted information on study and participant characteristics and conducted comprehensive subgroup and sensitivity analyses to identify potential sources of heterogeneity, explore the influence of study quality on pooled estimates, and assess the robustness of our results.

Although we sought additional estimates from authors, our analysis was limited to information available from the included studies. Studies reported different measures of association, which may have influenced results [46], however there were no statistical differences between HRR, IRR, CIR, or OR-based estimates. Most studies only reported crude estimates without adjusting for potential confounders and none of the eight authors of these studies that were contacted provided adjusted estimates, meaning the HIV-RAI association may be over- or underestimated. Potential for unmeasured or residual sources of confounding such as ART use and other partner characteristics cannot be totally ruled out even in adjusted analysis [47, 48]. Additionally, there was some evidence from funnel plots of publication bias toward higher aRR study estimates, suggesting authors may have been more likely to include RAI in multivariate models if the RAI-HIV association was high. Nevertheless, the overall pooled cRR estimate was similar, albeit slightly lower, than overall pooled aRR in both the full dataset and the subset of four studies reporting both cRR and aRR, and within these studies individual cRRs and aRRs did not differ substantially. This suggests the pooled cRR may somewhat underestimate the association but that inclusion of aRRs from studies that only provided cRRs would not have substantially influenced the pooled aRR. Despite substantial heterogeneity across both cRR and aRR study estimates, our overall pooled estimates were fairly robust since the direction and magnitude of association were not particularly influenced by any study estimate in our leave-one-out sensitivity analysis, although pooled aRR were more sensitive to individual study estimates partly due to the smaller number of estimates available.

Our subgroup analysis of crude and adjusted estimates highlighted potential differences in the magnitude of the RAI-HIV association by world region and risk population, respectively. Pooled estimates for the subset of studies conducted among women in sub-Saharan Africa (pooled cRR = 1.2) and among high-risk women (pooled aRR = 1.7) were lower than overall pooled estimates and non-statistically different than the null. As we did not find consistent patterns by both world region and risk population across estimates of both cRR and aRR in subgroup analysis, these differences may be real or due to methodological issues.

On the one hand, the low RAI-HIV association in high-risk women may partly reflect their exposure to multiple competing risk factors such as high rates of STIs and genital ulcer diseases [49, 50], large numbers of commercial and/or high-risk partners [51–53], differential levels of partner ART use [54], and differential frequencies of ejaculation by sex act [55], which may all increase the HIV acquisition risk during RVI and dilute the difference between RVI and RAI and were not properly accounted for in confounder analyses within the studies. Differences in ART use of sexual partners may explain variation in pooled estimates as evidence suggests that women practising condomless RAI are at a substantially reduced risk of HIV acquisition when their male sexual partners are taking suppressive ART [48]. However, this could not be explored as no included studies reported levels of partner ART use. Bias towards the null could also have occurred among high-risk women if they were more likely to use condoms during RAI than RVI, although evidence from reviews suggests condom use during RAI is as much as or slightly less than during RVI [6, 7]. Our pooled cRR for the only two studies reporting URAI only was higher (pooled cRR = 3.3) than for studies reporting any RAI (pooled cRR = 1.3), thus overall pooled estimates of the HIV-RAI association following exposure to URAI may be higher than our current pooled estimates for RAI. It was therefore surprising that only one aRR estimate was adjusted for condom use. We could not further explore URAI for adjusted estimates as none were based on URAI. Differences in RAI frequency among study participants may also explain variation in the magnitude of the RAI-HIV association by world region and risk populations [44], however this could not be investigated as no included studies reported RAI frequency.

On the other hand, exposure misclassification within original studies due to social desirability bias may explain regional variation in cRR estimates, since most studies recorded sexual behaviour data using FTFIs. RAI is highly stigmatised in many countries, particularly in sub-Saharan Africa, where it is also often misunderstood [56, 57]. For example, previous analyses of the ASPIRE and VOICE trials in Sub-Saharan Africa have reported contrasting levels of RAI reporting by women with FTFI (2%) compared to ACASI (17%) [58, 59]. A systematic review of heterosexual AI practice among South Africans similarly found higher reporting of lifetime AI with ACASI (pooled proportion = 29%) than FTFI (pooled proportion = 3%) [6]. Counterintuitively, our pooled cRR of the HIV-RAI association from the single study (reporting three independent estimates) using ACASI was lower than from studies using FTFI, which may reflect the greater risk of misinterpretation with ACASI [57]. Discussion of RAI is particularly taboo in some cultures, with some local languages such as Zulu (South Africa) and Shona (Zimbabwe) having no word for RAI or instead referring to it in only euphemistic terms [57], meaning questions regarding RAI are more likely to be misinterpreted as meaning RVI, but in a different position [57, 60, 61]. Therefore ACASI, although limiting social desirability bias, may not always more accurately estimate RAI than FTFI unless worded more carefully and pictorially. This could not be explored for adjusted estimates as none were based on ACASI.

Misclassification due to poor exposure definition was also possible [62, 63], and may have been more likely in studies defining RAI exposure at baseline and ever over a subject’s lifetime rather than exposure specifically during follow-up, as women reporting baseline RAI may not have practised it during follow-up [64]. Misclassification may also have occurred if ejaculation was not included in the RAI definition as women may not identify anal penetration without ejaculation as anal intercourse [65]. Additionally, since few studies reporting RAI during follow-up analysed it as time-varying, misclassification may have occurred as most studies did not account for intermittent RAI practice and because defining a precise recall period that coincides with the exact HIV exposure period is challenging since it depends on the testing frequency and the window period of the test [66–68]. Nonetheless, we did not find any differences by measurement of RAI in subgroup analysis.

Despite key limitations of original studies, including non-confidential interview methods and the lack of adjustment for potential confounders, most studies scored highly on the NOS, however the small number of included studies limited our own analysis. Even though few characteristics were identified that significantly influenced results in univariate subgroup analysis, we were unable to conduct multivariate analysis to explore factors that could explain the differential association by world region or risk population. Therefore, it is unclear whether the differences are real or the result of ecological bias due to the same set of studies having different characteristics.

Nonetheless, the findings of this review have public health implications for the prevention of HIV among women. The high cRR and aRR estimates from studies conducted before and after the introduction of ART in 1996 suggest that women who practise RAI remain at increased risk of HIV acquisition. RAI should no longer be neglected as a key risk factor and women should be provided with the means to prevent HIV acquisition through both RAI and RVI. This includes improving prevention messaging promoting condom use and oral PrEP, which are effective during both RAI and RVI, and developing new prevention technologies. Oral PrEP has proved effective in reducing individual acquisition risk in placebo-controlled trials when used appropriately, however uptake and adherence in trials has been limited and outside of trial settings PrEP is not yet widely used [57, 69]. Microbicides provide an alternative method of prevention, however vaginal microbicides have shown limited efficacy and would not be effective during RAI [9, 58, 69–72]. Dual compartment rectal and vaginal products could provide an important solution to this problem and recent studies suggest vaginally administered products may be able to provide dual protection during both RAI and RVI. For example, recent studies of intravaginal rings and vaginally-administered gels have demonstrated rapid dissemination of tenofovir and emtricitabine from vaginal to rectal tissue [73–75]. The continued development of such products is important to meet the need for greater choice in prevention methods, so women at risk of HIV acquisition are able to protect themselves.

A frequent limitation of studies included in our review was the poor methods used to quantify RAI practice or calculate RRs. It is surprising that after so many years and with the recognition of RAI as an important risk factor for HIV that RAI has not been more systematically investigated. Furthermore, with the knowledge that URAI is important for HIV transmission [3], the lack of data on URAI and condom use during RAI is disappointing. More longitudinal studies are urgently needed to evaluate whether the variation in RR by world region and risk population that we found is real and to provide further measures of RAI and condomless RAI. Such studies should define RAI more precisely including whether ejaculation occurred and test definitions to determine the extent to which women can report on ejaculation. They should standardise outcome measures to aid comparability across studies, and take into account condom use and RAI frequency. They should routinely measure RAI at regular intervals when HIV testing and carefully define the recall period to maximise the chance that it corresponds with the HIV exposure period prior to HIV acquisition. They should include RAI as a time-varying covariate in survival analyses to estimate RRs, and where possible include RAI in multivariate models with other important risk factors including condom use. Finally, confidential interview methods such as ACASI should also be used to limit social desirability bias and questions about RAI should be clear and unambiguous, potentially making use of visual aids, to minimise misinterpretation and to produce more accurate estimates of RAI practice [76, 77].

## Conclusions

In conclusion, women practising RAI may be at an increased risk of HIV acquisition, however there is some uncertainty in differences by region and risk population. Better data on RAI practice among women is necessary in order to answer the questions that this review has raised regarding variation in RAI-HIV risk by world region and risk population and to address gaps in the data surrounding the frequency of RAI and condom use during RAI. More prevention tools that provide dual protection against HIV acquisition during both RAI and RVI are needed, and women should be provided greater choice in prevention methods.

## Electronic supplementary material

Below is the link to the electronic supplementary material.

Supplementary material 1 (DOCX 356 kb)
