# Supplementary material for: Increases in HIV Incidence Following Receptive Anal Intercourse Among Women: A Systematic Review and Meta-analysis
Source: AIDS Behav. 2019 Sep 4;24(3):667–81. doi: 10.1007/s10461-019-02651-0 (PMC7018785; doi:10.1007/s10461-019-02651-0)
Supplement: Supplementary file 1 — Supplementary material 1 (DOCX 356 kb) [file 10461_2019_2651_MOESM1_ESM.docx]

**SUPPLEMENTARY MATERIALS**

**SUPPLEMENTARY METHODS**

**A. Embase search strategy**

| S1. Embase search strategy  Search conducted 03/09/18 |
| --- |
| ***Incidence domain*** (Longitudinal study/ OR Prospective study/ OR Cohort analysis/ OR (Cohort adj (study OR studies)).ab,ti. OR (follow up adj (study OR studies)).ab,ti. OR (observational adj (study OR studies)).ab,ti. OR incidence/ OR inciden*.ab,ti. OR ((rate* adj3 (ratio* OR inciden* OR hazard*)) OR (risk* adj3 (behavio* OR ratio* OR rate))).ab,ti. OR infection risk/ OR Randomized controlled trial/ OR Controlled clinical study/ OR human experiment/ OR (longitudin* OR prospective OR inciden* OR case control OR crossover OR cross over OR (controlled adj7 (study OR design OR trial))).ti,ab. OR Trial.ti. OR double blind procedure/ OR crossover procedure/ OR factorial design/ OR single blind procedure/ OR comparative study/ OR causality/ OR incidence/ OR infection rate/ OR survival rate/ OR cohort analysis/ OR case control study/ OR control group/ OR intention to treat analysis/ OR pretest posttest control group design/ OR pretest posttest design/ OR validation study/ OR panel study/ OR prevention study/ OR quasi experimental study/ OR experimental study/ OR evaluation study/ OR placebo.ti,ab. OR seroconv*.ab,ti. OR sero conv*.ab,ti. OR seroconversion/ OR serodiscordan*.ab,ti. OR sero discordan*.ab,ti.) NOT (qualitative OR (cross-sectional OR cross sectional)).ti. |
| **AND *women domain*** (female/ OR female*.ab,ti. OR wom#n.ab,ti.) |
| **AND *HIV domain*** (exp Human immunodeficiency virus/ OR HIV.ab,ti. OR (hiv-1* OR hiv-2* OR hiv1* OR hiv2*).ab,ti. OR hiv infect*.ab,ti. OR human immunodeficiency virus.ab,ti. OR human immunedeficiency virus.ab,ti. OR human immuno-deficiency virus.ab,ti. OR human immune-deficiency virus.ab,ti. OR (human immun* AND deficiency virus).ab,ti. OR acquired immunodeficiency syndrome.ab,ti. OR acquired immunedeficiency syndrome.ab,ti. OR acquired immuno-deficiency syndrome.ab,ti. OR acquired immune-deficiency syndrome.ab,ti. OR (acquired immun* AND deficiency syndrome).ab,ti.) |
| **AND *sexual behaviours domain*** (sexual behavior/ OR sexual intercourse/ OR (Sex* AND (act* OR intercourse OR behavio?r* OR anal OR risk factor*)).ab,ti.) |
| **NOT** ((rat OR rats OR mouse OR mice OR swine OR porcine OR murine OR sheep OR lambs OR pigs OR piglets OR rabbit OR rabbits OR cat OR cats OR dog OR dogs OR cattle OR bovine OR monkey OR monkeys OR trout OR marmoset$1).ti. AND animal experiment/) OR (Animal experiment/ NOT (human experiment/ OR human/) |
| **AND limit to yr="1980 -Current"** |

**B. Medline search strategy**

| S2. Medline search strategy  Search conducted 03/09/18 |
| --- |
| ***Incidence domain*** (Epidemiologic studies/ OR exp cohort studies/ OR (cohort adj (study OR studies)).ab,ti. OR cohort analy$.ab,ti. OR (follow up adj (study OR studies)).ab,ti. OR (observational adj (study OR studies)).ab,ti. OR longitudinal.ab,ti. OR prospective.ab,ti. OR Risk/ OR Incidence/ OR inciden*.ab,ti. OR ((rate* adj3 (behavio* OR ratio* OR inciden* OR hazard*)) OR (risk* adj3 (behavio* OR ratio* OR rate))).ab,ti. OR (longitudin* OR prospective OR inciden* OR case control OR crossover OR cross over OR (controlled adj7 (study OR design OR trial))).ti,ab. OR Trial.ti. OR Randomized Controlled Trials as Topic/ OR Controlled Clinical Trial/ OR Human Experimentation/ OR Double-Blind Method/ OR Cross-Over Studies/ OR Single-Blind Method/ OR Comparative Study/ OR Causality/ OR Survival Rate/ OR Cohort Studies/ OR Case-Control Studies/ OR Control Groups/ OR Intention to Treat Analysis/ OR Validation Studies/ OR Non-Randomized Controlled Trials as Topic/ OR Evaluation Studies/ OR placebo.ti,ab. OR seroconv*.ab,ti. OR sero conv*.ab,ti. OR Seroconversion/ OR serodiscordan*.ab,ti. OR sero discordan*.ab,ti.) NOT (qualitative OR (cross-sectional OR cross sectional)).ti. |
| **AND *women domain*** (Women/ OR Female/ OR wom#n.ab,ti. OR female*.ab,ti.) |
| **AND *HIV domain*** (exp HIV/ OR acquired immunodeficiency syndrome/ OR Hiv.ab,ti. OR hiv-1*.ab,ti. OR hiv-2*.ab,ti. OR hiv1.ab,ti. OR hiv2.ab,ti. OR hiv infect*.ab,ti. OR human immunodeficiency virus.ab,ti. OR human immunedeficiency virus.ab,ti. OR human immuno-deficiency virus.ab,ti. OR human immune-deficiency virus.ab,ti. OR (human immun* AND deficiency virus).ab,ti. OR acquired immunodeficiency syndrome.ab,ti. OR acquired immunedeficiency syndrome.ab,ti. OR acquired immuno-deficiency syndrome.ab,ti. OR acquired immune-deficiency syndrome.ab,ti. OR (acquired immun* AND deficiency syndrome).ab,ti.) |
| **AND *sexual behaviours domain*** (Sexual Behavior/ OR (Sex* AND (act* OR intercourse OR behavio?r* OR anal OR risk factor*)).ab,ti.) |
| **NOT** ((rat OR rats OR mouse OR mice OR swine OR porcine OR murine OR sheep OR lambs OR pigs OR piglets OR rabbit OR rabbits OR cat OR cats OR dog OR dogs OR cattle OR bovine OR monkey OR monkeys OR trout OR marmoset$1).ti. AND Animal Experimentation/) OR (Animal Experimentation/ NOT (Human Experimentation/ OR Humans/) |
| **AND limit to yr="1980 -Current"** |

**C. Criteria for contacting authors**

Authors were contacted for additional estimates when the association between RAI and incident HIV had either:

(1) been investigated but no estimate was reported;

(2) not been investigated but an estimate could potentially be derived; or

(3) if only the crude or adjusted estimate was reported.

**D. Data extraction**

The following formulae were used to derive estimates of odds ratio (OR), cumulative incidence ratio (CIR), incidence rate ratio (IRR), and standard errors of relative risk estimates that were then used to recalculate 95% CIs of study estimates before pooling

1. Odds Ratio (OR)

$$OR=\frac{ad}{bc}$$

$$SE\left\{ \ln\left( OR \right) \right\}= \sqrt{\frac{1}{a}+\frac{1}{b}+\frac{1}{c}+\frac{1}{d}}$$

$$95\% CI=exp(\ln\left( OR \right)\pm1.96*SE\{\ln\left( OR \right)\}$$

2. Cumulative Incidence Ratio (CIR)

$$CIR=\frac{a*\left( c+d \right)}{\left( a+b \right)*c}$$

$$SE\left\{ \ln\left( CIR \right) \right\}= \sqrt{\frac{{(n}_{1}-a)/a}{n_{1}}+\frac{{(n}_{2}-c)/c}{n_{2}}}$$

$$95\% CI=exp(\ln\left( CIR \right)\pm1.96*SE\{\ln\left( CIR \right)\}$$

3. Incidence Rate Ratio (IRR)

$$IRR=\frac{{IR}_{1}}{{IR}_{0}}$$

$$SE\left\{ \ln\left( \mathrm{IRR} \right) \right\}= \sqrt{\frac{1}{a}+\frac{1}{c}}$$

$$95\% CI=exp(\ln\left( IRR \right)\pm1.96*SE\{\ln\left( IRR \right)\}$$

*a* = number of women that practiced RAI and acquired HIV

*b* = number of women that practiced RAI and did not acquire HIV

*c* = number of women that did not practice RAI and acquired HIV

*d* = number of women that did not practice RAI and did not acquire HIV

*n_1_* = total number of women that practiced RAI

*n_2_* = total number of women that did not practice RAI

*IR_1_* = incidence rate of HIV in women that practiced RAI

*IR_2_* = incidence rate of HIV in women that did not practice RAI

1. Standard error of relative risk (RR) estimates

Lower 95% CI (RR) = ln(lower confidence limit given for RR)

Upper 95% CI (RR) = ln(upper confidence limit given for RR)

Effect estimate = lnRR

SE = (upper 95% CI – lower 95% CI) / 3.92

**E. Newcastle-Ottawa quality assessment scale criteria**

**Cohort studies and controlled trials:**

| **Criteria** | **Condition required to obtain a point** | **Bias assessed** |
| --- | --- | --- |
| Selection 1) Representativeness of the exposed cohort (HIV-infected) | Exposed individuals were representative of women in the community, or of an epidemiological core group (e.g. FSW, SDCs) | Sample representativeness |
| Selection 2) Selection of the non-exposed cohort | Non-exposed women were chosen from the same community as exposed women. Awarded for all studies. | Differential selection on basis of exposure |
| Selection 3) Ascertainment of exposure | Exposure information collected using confidential interview methods (e.g. ACASI) over non-confidential methods (e.g. FTFI) | Misclassification of exposure |
| Selection 4) Demonstration that outcome of interest was not present at start of study | Awarded for all studies as testing was a criterion for inclusion and all studies were longitudinal | Misclassification of outcome |
| Comparability 1a) Comparability of cohorts on the basis of the design or analysis | Adjustment or matching for age was done | Confounding |
| Comparability 1b) Comparability of cohorts on the basis of the design or analysis | Adjustment or matching for other potential important confounders including condom use, partner ART use, or STIs was done | Confounding |
| Outcome 1) Assessment of outcome | Method for testing was stated | Misclassification of outcome |
| Outcome 2) Was follow-up long enough for outcomes to occur | Follow-up of adequate length (>6 months) | Inadequate assessment of outcome |
| Outcome 3) Adequacy of follow-up cohorts | Follow-up rate at least 60% | Loss-to-follow up |

**SUPPLEMENTARY RESULTS**

**Table S1. Table of additional information on included studies.**

Further details on participant and study characteristics of studies included in the meta-analysis that reported crude and adjusted relative risks of the association between RAI practice and HIV incidence among women

|  | **Number of studies (N_s_=17)** | **Crude RR** | | **Adjusted RR** | |
| --- | --- | --- | --- | --- | --- |
| **Variable** |  | **Number of estimates (N_e_=18)** | **References** | **Number of estimates (N_e_=5)** | **References** |
| **Participant characteristics** |  |  |  |  |  |
| World region |  |  |  |  |  |
| Africa | 12 | 13 | (12,14,15,28–35) | 4 | (31,33,36,37) |
| Out of Africa | 5 | 5 | (13,38–41) | 1 | (42) |
| Risk population |  |  |  |  |  |
| General-risk | 6 | 7 | (15,28,30,34,39) | 1 | (36) |
| High-risk | 11 | 11 | (12–14,29,31–33,35,38,40,41) | 4 | (31,33,37,42) |
| Mean or median age^†^ |  |  |  |  |  |
| ≤28 years | 10 | 7 | (12,13,28,29,32,33,35,40,41) | 3 | (33,36,37) |
| >28 years | 6 | 6 | (14,15,31,34,39) | 2 | (31,42) |
| NR | 2 | 5 | (30,38) | 0 | - |
| RAI prevalence^†^ |  |  |  |  |  |
| ≤14% | 8 | 8 | (12,13,15,28,30,34,40) | 1 | (36) |
| >14% | 7 | 7 | (14,29,31,34,35,39,41) | 2 | (31,37) |
| NR | 3 | 3 | (32,33,38) | 2 | (33,42) |
| **Study characteristics and quality** | | | | | |
| Study year^‡^ |  |  |  |  |  |
| Pre-1996 | 6 | 6 | (35,38–41) | 1 | (42) |
| 1996 onwards | 11 | 12 | (12–15,28–34) | 4 | (31,33,36,37) |
| Study design |  |  |  |  |  |
| Cohort | 9 | 9 | (28,29,31,34,35,38,39) | 3 | (31,36,37) |
| RCT | 6 | 7 | (12–15,30,32,33) | 1 | (33) |
| Serodiscordant couple | 2 | 2 | (40,41) | 1 | (42) |
| Interview method |  |  |  |  |  |
| ACASI | 1 | 3 | (34) | 0 | - |
| FTFI | 16 | 15 | (12–15,28–33,35,38–41) | 5 | (31,33,36,37,42) |
| Measurement of exposure^†§^ |  |  |  |  |  |
| Baseline: short time-frame | 2 | 2 | (28,32) | 0 | - |
| Baseline: long time-frame | 4 | 3 | (13,31,35) | 2 | (31,36) |
| During follow-up: anytime | 7 | 7 | (12,15,29,30,39–41) | 0 | - |
| During follow-up: time-varying | 4 | 5 | (33,34,38) | 2 | (33,42) |
| Unclear | 1 | 1 | (14) | 1 | (37) |
| Definition of RAI |  |  |  |  |  |
| URAI only | 2 | 2 | (13,15) | 0 | - |
| Any RAI | 15 | 16 | (12,14,15,28–35,38–41) | 5 | (31,33,36,37,42) |
| Type of measure^†^ |  |  |  |  |  |
| HRR | 8 | 7 | (14,32–34,38) | 5 | (31,33,36,37,42) |
| IRR | 6 | 6 | (12,29,30,40,41) | 0 | - |
| CIR | 4 | 4 | (13,15,28,35) | 0 | - |
| OR | 1 | 1 | (39) | 0 | - |
| Extraction of estimate^†^ |  |  |  |  |  |
| Directly reported | 13 | 13 | (12–14,29,31,32,34,28–41) | 5 | (31,33,36,37,42) |
| Self-calculated/from authors | 5 | 5 | (15,28,30,33,35) | 0 | - |
| Location RAI first reported^†^ |  |  |  |  |  |
| Title/abstract | 5 | 5 | (13,29,39–41) | 0 | - |
| Text/table | 13 | 13 | (12,14,15,28,30–35,38) | 5 | (31,33,36,37,42) |
| ACASI, audio computer-assisted self-interview; CI, confidence interval; CIR, cumulative incidence ratio; FTFI, face-to-face interview; IRR, incidence rate ratio; HRR, hazard rate ratio; N_e_, number of estimates; NR, not reported; N_s_, number of studies; OR, odds ratio; RAI, receptive anal intercourse; RCT, randomised controlled trial; RR, relative risk; URAI, unprotected (condomless and no PrEP) receptive anal intercourse  Continuous variables were dichotomised at the median, except for study year , which was dichotomised at the boundary between the pre-, and post-antiretroviral treatment (ART) eras (1996).  † same study included in more than one subcategory  ‡ study year is the year at the midpoint of the study  § short time frame includes RAI in the past 6 months or less, long time frame includes RAI in the past year to lifetime | | | | | |

**Table S2.** Results of quality assessment analysis of studies included in the meta-analysis of cRR and aRR using Newcastle-Ottawa Quality Assessment Scale for Cohort Studies

| **Newcastle-Ottawa Quality Assessment Scale for Cohort Studies** | | | | | | | | | |
| --- | --- | --- | --- | --- | --- | --- | --- | --- | --- |
| **Reference** | **Selection** (max. 4 points) | | | | **Comparability** (max. 2 points) | **Outcome** (max. 3 points) | | | **Total** (max. 9 points) |
|  | Representativeness of the exposed cohort | Selection of the non-exposed cohort | Ascertainment of exposure | Demonstration that outcome of interest was not present at start of study | Comparability of cohorts on the basis of the design or analysis | Assessment of outcome | Was follow-up long enough for outcomes to occur | Adequacy of follow-up cohorts |  |
| **CRUDE ESTIMATES** | | | | | | | | | |
| Auvert 2011 (13) | 0 | 1 | 0 | 1 | 0 | 1 | 1 | 1 | 5 |
| Chirgwin 1999 (39) | 1 | 1 | 0 | 1 | 0 | 1 | 1 | 1 | 6 |
| de Vincenzi 1994 (40) | 1 | 1 | 0 | 1 | 0 | 1 | 1 | 1 | 6 |
| Dong 2018 (28) | 1 | 1 | 0 | 1 | 0 | 1 | 1 | 1 | 6 |
| Feldblum 2010 (32) | 1 | 1 | 0 | 1 | 0 | 1 | 1 | 1 | 6 |
| Ghys 2001 (12) | 1 | 1 | 0 | 1 | 0 | 1 | 1 | 0 | 5 |
| Kilmarx 1998 (38) | 1 | 1 | 0 | 1 | 0 | 1 | 1 | 1 | 6 |
| Laga 1993 (35) | 1 | 1 | 0 | 1 | 0 | 1 | 1 | 1 | 6 |
| Mavedzenge 2011 (34) | 1 | 1 | 1 | 1 | 0 | 1 | 1 | 1 | 7 |
| McCormack 2010 (30) | 1 | 1 | 0 | 1 | 0 | 1 | 1 | 1 | 6 |
| Naicker 2015 (31) | 1 | 1 | 0 | 1 | 0 | 1 | 1 | 0 | 5 |
| Novak 2013 (13) | 1 | 1 | 0 | 1 | 0 | 1 | 1 | 1 | 6 |
| Priddy 2011 (29) | 0 | 1 | 0 | 1 | 0 | 1 | 1 | 1 | 5 |
| Saracco 1993 (41) | 1 | 1 | 0 | 1 | 0 | 1 | 1 | 1 | 6 |
| Skoler-Karpoff 2008 (15) | 1 | 1 | 0 | 1 | 0 | 1 | 1 | 1 | 6 |
| Watson-Jones 2009 (33) | 0 | 1 | 0 | 1 | 0 | 1 | 1 | 1 | 5 |
| **ADJUSTED ESTIMATES** | | | | | | | | | |
| Nel 2011 (36) | 0 | 1 | 0 | 1 | 1 | 1 | 1 | 1 | 6 |
| Naicker 2015 (31) | 1 | 1 | 0 | 1 | 2 | 1 | 1 | 0 | 7 |
| Ramjee 2005 (37) | 0 | 1 | 0 | 1 | 1 | 1 | 1 | 1 | 6 |
| Saracco 1997 (42) | 1 | 1 | 0 | 1 | 2 | 1 | 1 | 1 | 8 |
| Watson-Jones 2009 (33) | 0 | 1 | 0 | 1 | 1 | 1 | 1 | 1 | 6 |

**Table S3.** Table showing pooled estimates of adjusted relative risks estimates in subgroup analyses of study estimates stratified by participant and study characteristics and quality indicators.

| **Variable** | **N_e_** | **References** | **Pooled aRR** | **95% CI** | **p-value** | **I^2^** |
| --- | --- | --- | --- | --- | --- | --- |
| **A. Participant characteristics** | | | | | | |
| Region |  |  |  |  | 0.90 |  |
| Africa | 4 | (31,33,36,37) | 2.29 | 0.82 – 6.42 | - | 74% |
| Out of Africa | 1 | (42) | 2.50 | 1.12 – 5.59 | - | - |
| Risk population |  |  |  |  | 0.06 |  |
| General-risk | 1 | (36) | 8.50 | 1.90 – 38.0 | - | 0% |
| High-risk | 4 | (31,33,37,42) | 1.69 | 0.82 – 3.47 | - | 63% |
| Mean age |  |  |  |  | 0.65 |  |
| ≤28 | 3 | (33,36,37) | 3.14 | 0.52 – 18.9 | - | 82% |
| >28 | 2 | (31,42) | 2.04 | 1.15 – 3.61 | - | 0% |
| **B. Study characteristics and quality** | | | | | | |
| Study year^†^ |  |  |  |  | 0.90 |  |
| Pre-1996 | 1 | (42) | 2.50 | 1.12 – 5.59 | - | - |
| 1996 onwards | 4 | (31,33,36,37) | 2.29 | 0.82 – 6.42 | - | 76% |
| Measurement of exposure |  |  |  |  | 0.33 |  |
| Baseline: long time frame | 2 | (31,36) | 3.31 | 0.68 – 16.2 | - | 72% |
| During follow-up: time-varying | 2 | (33,42) | 2.88 | 1.37 – 6.08 | - | 0% |
| Unclear | 1 | (37) | 0.82 | 0.46 – 1.44 | - | - |
| Study design |  |  |  |  | 0.53 |  |
| Cohort | 3 | (31,36,37) | 1.85 | 0.63 – 5.45 | - | 77% |
| RCT | 1 | (33) | 6.87 | 0.94 – 50.5 | - | - |
| Serodiscordant couple | 1 | (42) | 2.50 | 1.12 – 5.59 | - | - |
| NOS score |  |  |  |  | 0.65 |  |
| ≤6 | 3 | (33,36,37) | 3.14 | 0.52 – 18.9 | - | 82% |
| >6 | 2 | (31,42) | 2.04 | 1.15 – 3.61 | - | 0% |
| aRR, adjusted relative risk; CI, confidence interval; N_e_, number of estimates; NOS, Newcastle-Ottawa Scale; NR, not reported; RCT, randomised controlled trial  Continuous variables were dichotomised at the median, except for study year , which was dichotomised at the boundary between the pre-, and post-antiretroviral treatment (ART) eras (1996).  † Study year is the midpoint between study start and finish | | | | | | |

**.**

**Figure S1.** Forest plot of crude (cRR, blue) and adjusted (aRR, red) study estimates of relative risk from the subset of four studies reporting estimates of both, and their corresponding pooled estimates (diamonds).**
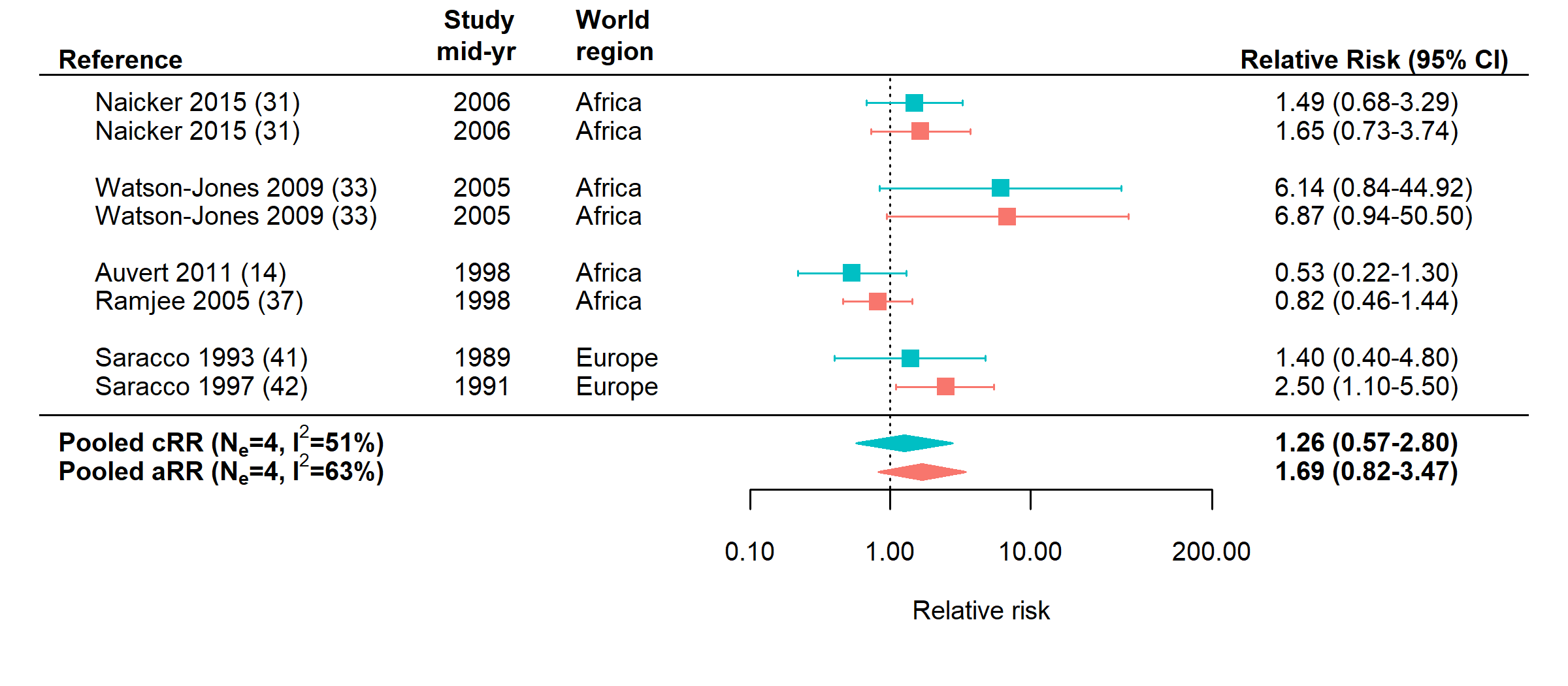
**

**Figure S2. Funnel plots.**

A) Crude (blue) and B) adjusted (red) relative risks of the RAI-HIV association are plotted against their standard errors. Pooled estimates (solid vertical lines) and pseudo 95% CIs (the area within which 95% of study estimates should lie in the absence of bias or heterogeneity) (dashed lines) are also shown. There was some evidence of publication bias across study estimates of aRR.

**A).** Funnel plot of cRR estimates

***
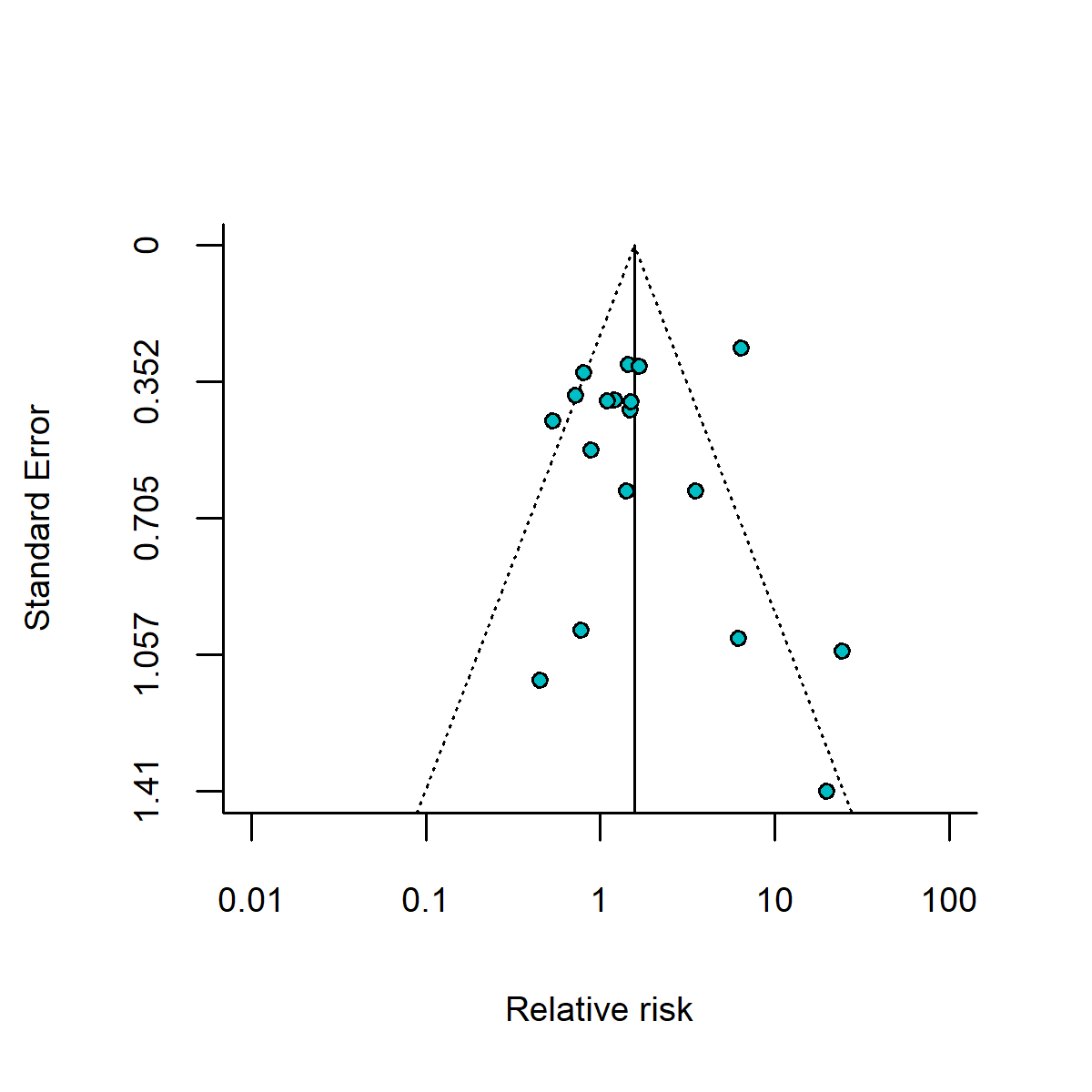
***

**B).** Funnel plot of aRR estimates

**
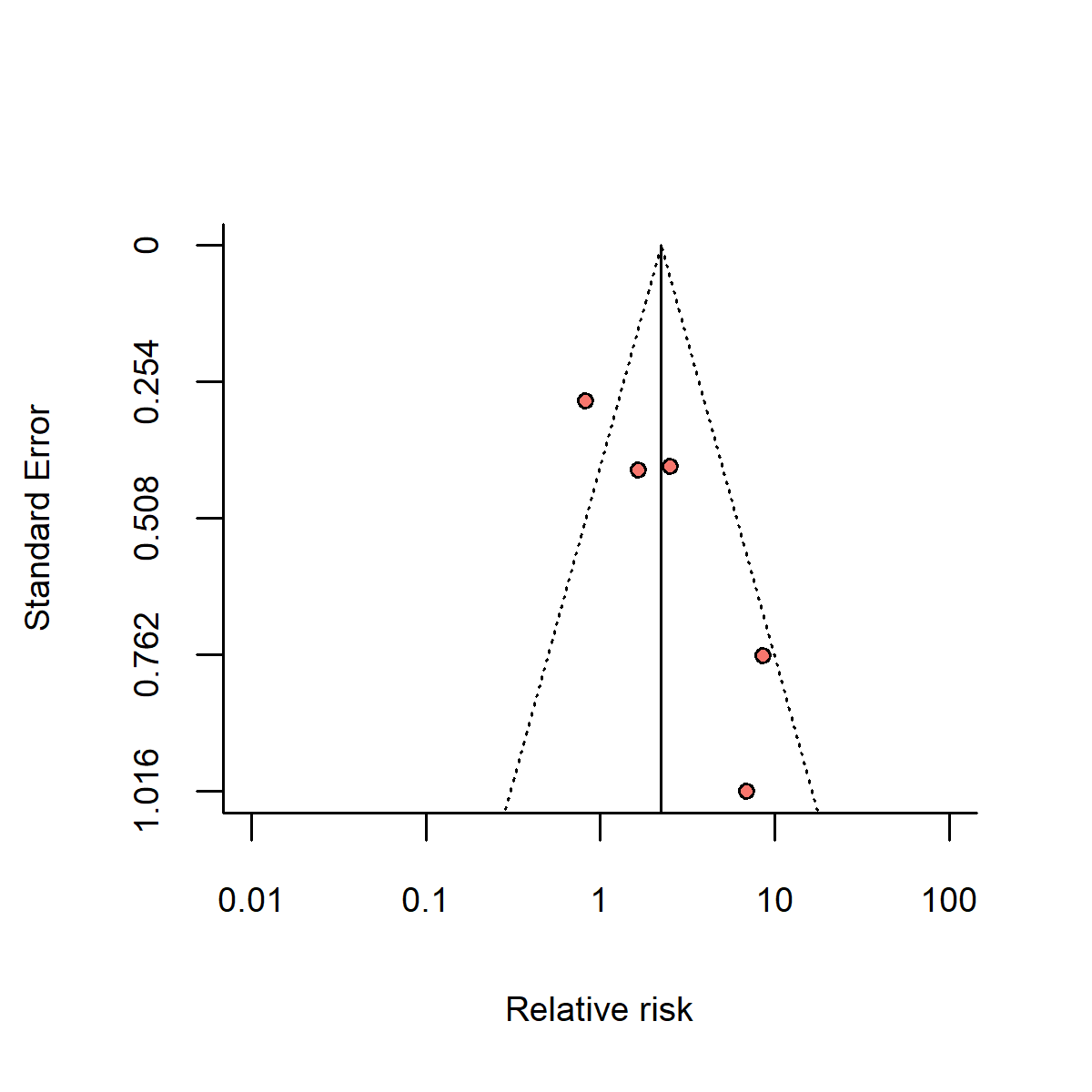
Figure S3. Leave-one-out sensitivity analysis.**

The forest plots show pooled crude relative risk (cRR, blue) estimates (A) overall, and (B) in Africa only, and pooled adjusted relative risk (aRR, red) estimates (C) overall. Pooled estimates and their 95% CIs were calculated through sequentially omitting individual study estimates from the overall pooled estimate. The omitted estimate is named on the y-axis. The overall pooled estimates and 95% CIs including all studies are shown as vertical solid and dashed lines, respectively.

**A).** Sensitivity analysis of study estimates of cRR


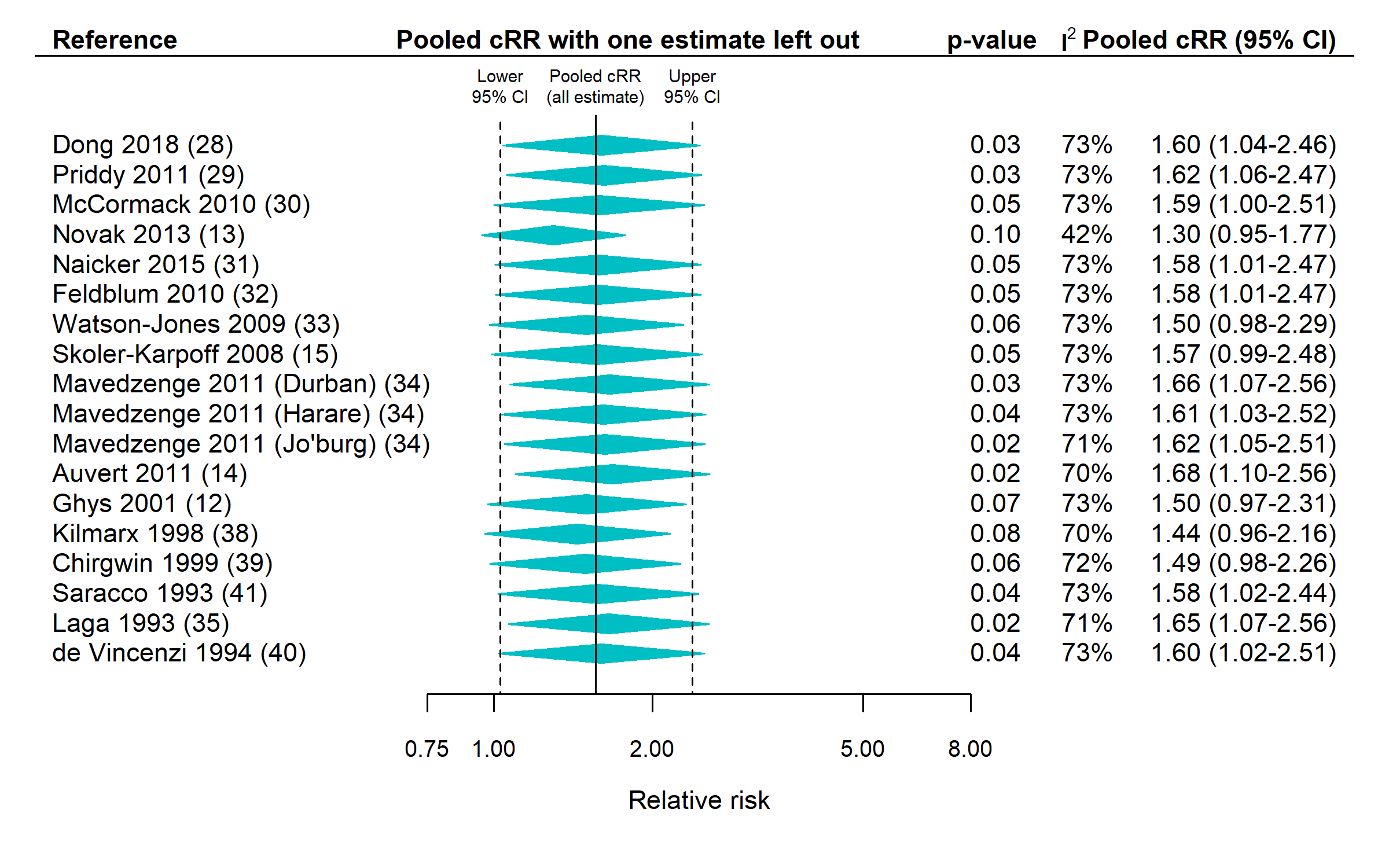
**B).** Sensitivity analysis of crude estimates from studies conducted in Africa


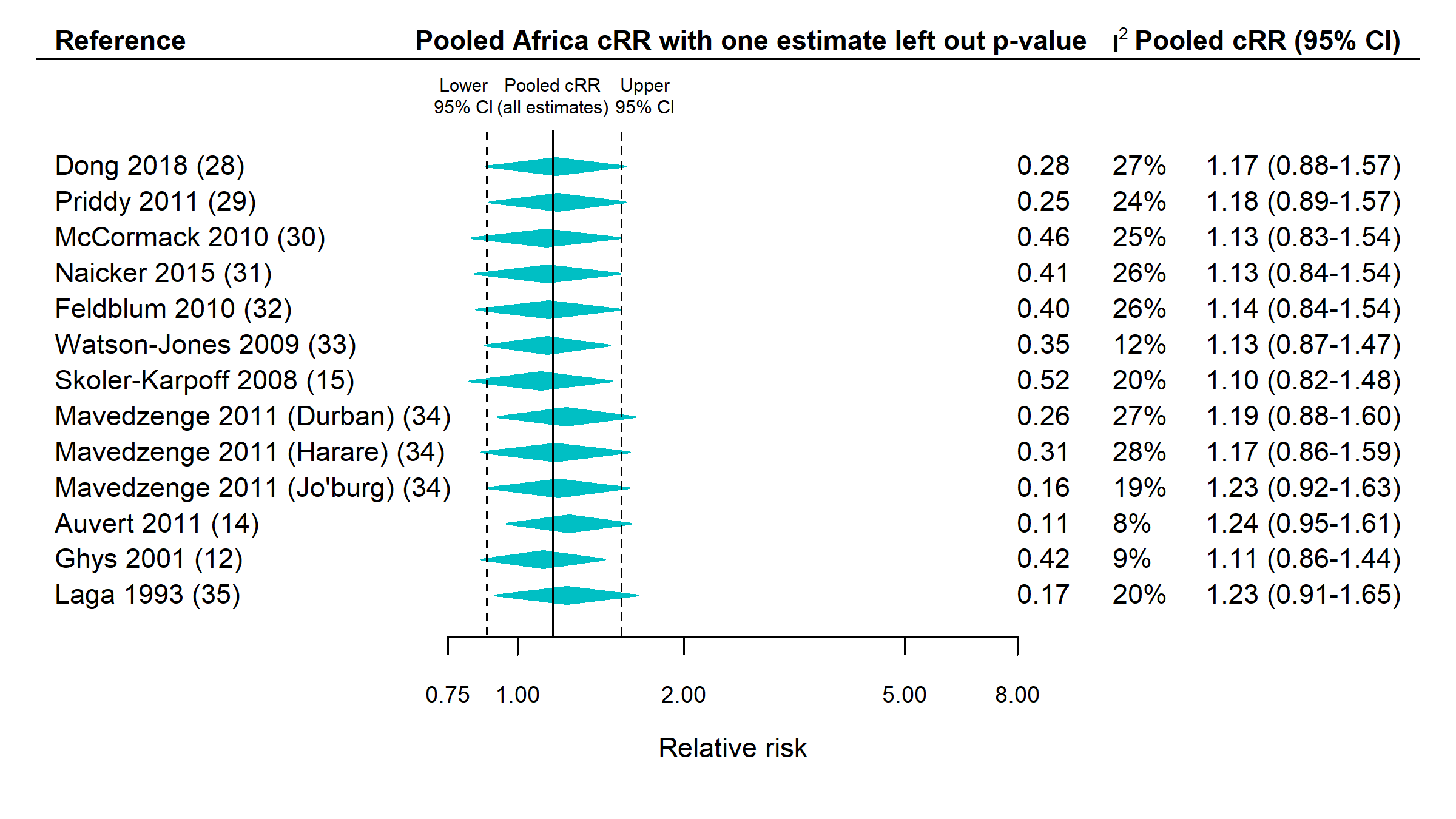


**C).** Sensitivity analysis of study estimates of aRR

**
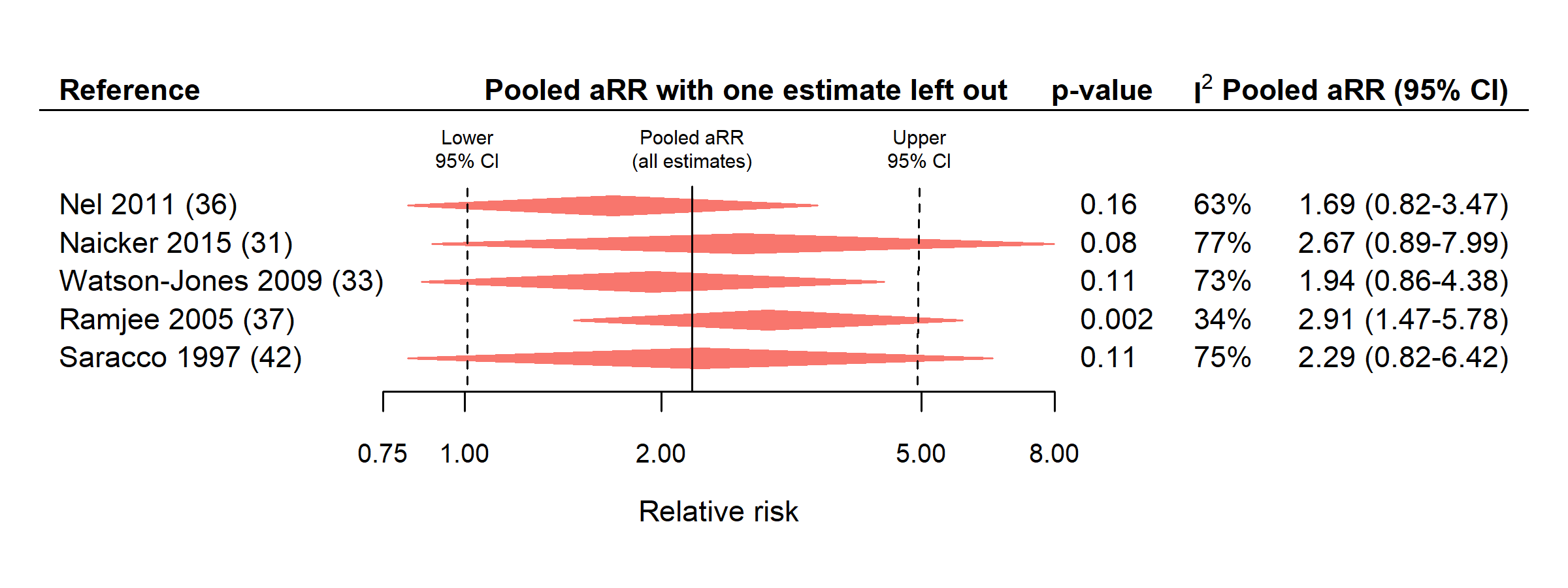
**
